# Escalating costs of self-injury mortality in the 21st century United States: an interstate observational study

**DOI:** 10.1186/s12889-023-15188-8

**Published:** 2023-02-08

**Authors:** Ian R.H. Rockett, Bina Ali, Eric D. Caine, Donald S. Shepard, Aniruddha Banerjee, Kurt B. Nolte, Hilary S. Connery, G. Luke Larkin, Steven Stack, Franklin M.M. White, Haomiao Jia, Jeralynn S. Cossman, Judith Feinberg, Amanda N. Stover, Ted R. Miller

**Affiliations:** 1 Department of Epidemiology and Biostatistics, West Virginia University School of Public Health, One Medical Center Drive, Morgantown, WV 26506-9190 USA; 2 Department of Psychiatry, University of Rochester Medical Center, 300 Crittenden Blvd, Rochester, NY 14642 USA; 3 Pacific Institute for Research and Evaluation, 4061 Powder Mill Rd, Beltsville, MD 20705 USA; 4 Cost and Value Group, Heller School for Social Policy and Management, Brandeis University, 415 South St, Waltham, MA 02453 USA; 5 Department of Geography, Indiana University-Purdue University at Indianapolis, Cavanaugh Hall 441, 425 University Blvd., Indianapolis, IN 46202 USA; 6 Department of Pathology, University of New Mexico School of Medicine, MSC08-4640, Albuquerque, NM 87131 USA; 7 McLean Hospital, 115 Mill Street, Mail Stop 222, Belmont, MA 02478-1064 USA; 8 Department of Psychiatry, Harvard Medical School, 2 West, Room 305, 401 Park Drive, Boston, MA 02215 USA; 9 Northeast Ohio Medical University, 4209 St. Rt. 44, PO Box 95, Rootstown, OH 44272 USA; 10 Department of Criminology and Criminal Justice, Wayne State University, 3293 Faculty/Administration Building (FAB) 656 W. Kirby St, Detroit, MI 48202 USA; 11 Department of Psychiatry and Behavioral Neurosciences, Wayne State University, Tolan Park Medical Building, 3901 Chrysler Service Drive, Detroit, MI 48201-2167 USA; 12 Department of Community Health and Epidemiology, Dalhousie University, 5790 University Ave, Halifax, NS B3H 1V7 Canada; 13 Department of Biostatistics, Mailman School of Public Health, Columbia University, 722 W 168th St, New York, NY 10032 USA; 14 School of Nursing, Columbia University, 560 W 168th St, New York, NY 10032 USA; 15 College for Health, Community and Policy, University of Texas-San Antonio, One UTSA Circle, San Antonio, TX 78249-3209 USA; 16 Departments of Behavioral Medicine and Psychiatry and Medicine, Infectious Diseases, West Virginia University School of Medicine, 30 Chestnut Ridge Rd, Morgantown, WV 26506 USA; 17 Eshelman School of Pharmacy, University of North Carolina at Asheville, One University Heights, 2214 Kerr Hall, Asheville, NC 28804 USA; 18 Centre for Population Health Research, Curtin University, 208 Kent St, Bentley, WA 6102 Australia

**Keywords:** Suicide, drug overdose, Cost, Poisoning, Injury prevention, Mortality, Drug dependence, Addiction

## Abstract

**Background:**

Estimating the economic costs of self-injury mortality (SIM) can inform health planning and clinical and public health interventions, serve as a basis for their evaluation, and provide the foundation for broadly disseminating evidence-based policies and practices. SIM is operationalized as a composite of all registered suicides at any age, and 80% of drug overdose (intoxication) deaths medicolegally classified as ‘accidents,’ and 90% of corresponding undetermined (intent) deaths in the age group 15 years and older. It is the long-term practice of the United States (US) Centers for Disease Control and Prevention (CDC) to subsume poisoning (drug and nondrug) deaths under the injury rubric. This study aimed to estimate magnitude and change in SIM and suicide costs in 2019 dollars for the United States (US), including the 50 states and the District of Columbia.

**Methods:**

Cost estimates were generated from underlying cause-of-death data for 1999/2000 and 2018/2019 from the US Centers for Disease Control and Prevention’s (CDC’s) Wide-ranging ONline Data for Epidemiologic Research (WONDER). Estimation utilized the updated version of Medical and Work Loss Cost Estimation Methods for CDC’s Web-based Injury Statistics Query and Reporting System (WISQARS). Exposures were medical expenditures, lost work productivity, and future quality of life loss. Main outcome measures were disaggregated, annual-averaged total and per capita costs of SIM and suicide for the nation and states in 1999/2000 and 2018/2019.

**Results:**

40,834 annual-averaged self-injury deaths in 1999/2000 and 101,325 in 2018/2019 were identified. Estimated national costs of SIM rose by 143% from $0.46 trillion to $1.12 trillion. Ratios of quality of life and work losses to medical spending in 2019 US dollars in 2018/2019 were 1,476 and 526, respectively, versus 1,419 and 526 in 1999/2000. Total national suicide costs increased 58%—from $318.6 billion to $502.7 billion. National per capita costs of SIM doubled from $1,638 to $3,413 over the observation period; costs of the suicide component rose from $1,137 to $1,534. States in the top quintile for per capita SIM, those whose cost increases exceeded 152%, concentrated in the Great Lakes, Southeast, Mideast and New England. States in the bottom quintile, those with per capita cost increases below 70%, were located in the Far West, Southwest, Plains, and Rocky Mountain regions. West Virginia exhibited the largest increase at 263% and Nevada the smallest at 22%. Percentage per capita cost increases for suicide were smaller than for SIM. Only the Far West, Southwest and Mideast were not represented in the top quintile, which comprised states with increases of 50% or greater. The bottom quintile comprised states with per capita suicide cost increases below 24%. Regions represented were the Far West, Southeast, Mideast and New England. North Dakota and Nevada occupied the extremes on the cost change continuum at 75% and − 1%, respectively.

**Conclusion:**

The scale and surge in the economic costs of SIM to society are large. Federal and state prevention and intervention programs should be financed with a clear understanding of the total costs—fiscal, social, and personal—incurred by deaths due to self-injurious behaviors.

## Introduction

Economic cost is the bottom line in measuring injury and disease burdens to society and justifying ameliorative policies, planning, and interventions. Traditionally, suicide has been the sole representative of fatal self-injury, The most recent (2013) estimate of the economic costs of suicide in the United States (US), in terms of medical spending and lost work productivity, but unadjusted for quality-of-life losses, approximated $61.5 billion [1] in 2019 US dollars; per capita cost was $1,536 [2]. Factoring all three of these elements into the calculations, a 2021 study from the Centers for Disease Control and Prevention (CDC) estimated the economic costs of total injury mortality in 2019 at $2.2 trillion [3]. A complementary report estimated that state per capita injury costs ranged from $4,538 for New York to $11,274 for West Virginia [4]. Derived from a third 2021 CDC study, confined to the District of Columbia (DC) and the 38 states then participating in the now universal National Violent Death Reporting System, the estimated cost in 2019 dollars [2] of fatal overdoses from opioids was $581 billion for the year 2017[5]. Corresponding per capita costs ranged from $453 for Hawaii to $5,560 for West Virginia.

Self-injury mortality (SIM) has been proposed as an alternative and broader representative of fatal self-injury than suicide alone [6, 7]. SIM merges registered or known suicides by any method with the preponderance of opioid, alcohol and other drug poisoning deaths [8]. It was founded on another novel concept, ‘death from drug self-intoxication,’ which is a rejection of the predominant notion that most overdose fatalities are ‘accidents’—the formal manner-of-death category to which medical examiners and coroners principally assign them [9]. Rather, these deaths reflect patterns of motivated self-harming behaviors related to the acquisition and use of potentially harmful substances, whether or not the decedents consciously intended to die on their respective days of death [10]. SIM is distinguished from a kindred concept, ‘deaths of despair’ [11], by restricting its domain to acute injury deaths reflecting decedents’ instrumental actions, while not including progressive, fatal diseases associated with alcohol (e.g., alcoholic cirrhosis or cardiomyopathy) or other drug use (e.g., HIV, hepatitis C, or endocarditis).

SIM has surpassed diabetes mellitus [12], kidney disease, and influenza and pneumonia as a killer of Americans, and accounts for a much higher proportion of premature mortality [13]. The ongoing crisis with SIM originated two decades prior to the onset of the COVID-19 pandemic [5]; the US is now afflicted by a major mental health crisis across all states that has accelerated steadily [8]. This situation contrasts with the more geographically limited picture associated with suicide, which taken by itself reflects a regional distribution most heavily tilted towards Western states. In broadening the scope of fatal self-injury, SIM mitigates differential misclassification biases and data acquisition challenges when comparing the relative difficulty of definitively identifying suicides related to drug poisoning to the relative ease of identifying suicides due to firearms and hanging [14, 15]. Exacerbating potential for such bias has been the rapid dissemination of highly lethal, synthetic opioid compounds [16]. SIM also mitigates downward biases in suicide identification that implicate biological sex, race/ethnicity [14], and likely, age and education [17], as well as the inconsistent availability of compelling psychiatric histories and forensic data [14, 17–19].

In this study, we estimate the magnitude and change in the economic costs of SIM and suicide for the states and nation between 1999/2000 and 2018/2019. Results harbor important implications for health planning, clinical and public health interventions, their evaluation, and evidence-based policy and practice.

## Methods

This cross-sectional study was a secondary analysis of an aggregated, state-level, publicly accessible mortality dataset. The study adhered to the Strengthening the Reporting of Observational Studies in Epidemiology (STROBE) reporting guideline.

### Data sources and SIM operationalization

SIM is based on respective annualized, state-level, deidentified manner and underlying cause-of-death data and associated population data for 1999/2000 and 2018/2019 for all 50 US states and DC from CDC’s Wide-ranging ONline Data for Epidemiologic Research (WONDER) [20]. State nosologists precoded certified deaths according to the International Statistical Classification of Diseases and Related Health Problems, Tenth Revision [21] prior to submission to the National Center for Health Statistics. This study subscribes to the long-term practice of CDC to subsume poisoning deaths (drug and nondrug) under the injury rubric. SIM comprised all suicides (ICD-10 UO3, X60-X84, Y 87.0) by any method, irrespective of decedent age, and 80% of ‘accidental’ (‘unintentional’ per CDC nomenclature) opioid, alcohol and other drug intoxication deaths (X40-X45) and 90% of corresponding drug intoxication deaths of undetermined intent (Y10-15) among persons ages 15 years and older. Following previous practice [8, 12, 13, 22], this SIM formulation excluded the small proportion of estimated drug intoxication deaths not attributable to repetitive self-harm behaviors commonly associated with substance use disorders (e.g., from fatal interactions between compliant use of prescription opioids with that of other medications). The rationale for the age cutoff for the ‘accidental’ and undetermined (intent) drug self-intoxication components of SIM was the assumed rarity of purposive self-harm behaviors among children and younger adolescents during the observation period [23].

### National and state cost calculations

Utilizing the updated version of Medical and Work Loss Cost Estimation Methods for CDC’s Web-based Injury Statistics Query and Reporting System (WISQARS) Cost of Injury Module [24], we estimated the economic costs of SIM and suicide for the nation, states and DC. The breakdown of the medical spending, lost work productivity, and future quality of life components of SIM in 1999/2000 and 2018/2019 are included in this report. Medical costs were assigned by place of death—on scene/at home, arrival at hospital, emergency department, hospital after admission, nursing home, or hospice. Deaths in a medical facility were assigned treatment costs by injury mechanism, age, and diagnosis using the mean estimated medical costs of injury deaths in nationally representative samples covering the respective facility type, where sample size permitted. Emergency transportation costs were included in all deaths except those occurring on scene/at home. Coroner or medical examiner costs were added if an autopsy was performed. Work loss costs included the estimated wages, fringe benefits, and household work over the expected remaining life span of the victim in the absence of premature death. We computed sex-specific work life costs over the expected remaining life span using life table probabilities of surviving from each year of age to the next and average annual earnings by age group. Average annual earnings implicitly account for both the probability of being employed, the hours worked if employed, and the wage rate.

Adopting a societal perspective, we factored in quality-of-life losses by monetizing the estimated quality-adjusted life years lost as detailed elsewhere [25–28]. We valued the lives lost with the $10.7 million average value per statistical life (VSL) that the US Department of Health and Human Services uses in regulatory analysis [29]. VSL is the value per life saved that results from aggregating across the population the amount that people pay (or state in surveys that they are willing to pay) for small reductions in their own or an immediate family member’s chance of dying [30]. The $10.7 million VSL comes from a meta-analysis of a large literature [31]. Subtracting lifetime work loss from VSL yields the value of lost quality of life. Quality of life encompasses all of life’s nonmonetary aspects, including a person’s health, comfort, and ability to participate in and enjoy social and role functions [32]. Dividing average lifetime quality of life loss by life expectancy yields a cost per year of quality of life lost that can be used to tailor losses by decedent age and sex [33]. Based on previous work that suggests reduced quality of life for individuals with substance use and mood disorders (e.g., 9-25% loss of quality of life for substance use disorders [34–37]; and 15-20% loss for people with mood disorders and suicide risk [38, 39], conservatively, we valued loss of a lifetime of quality of life from each SIM death at 80% of the loss for the average person of the same age and sex. Costs beyond the first year were discounted to present value using a widely recommended 3% annual discount rate [40]. We inflated all costs to 2019 dollars and used two-year annual-averaged rates and costs to stabilize the data. Costs previously cited from other reports were also expressed in 2019 US dollars, with extrapolation using multipliers where necessary [2], for comparability with our results. Where we differentiated regions, we employed the eight regions utilized by the US Bureau for Economic Analysis: Far West, Rocky Mountain, Plains, Great Lakes, New England, Mideast, Southeast and Southwest [41].

## Results

**Fig. 1:**
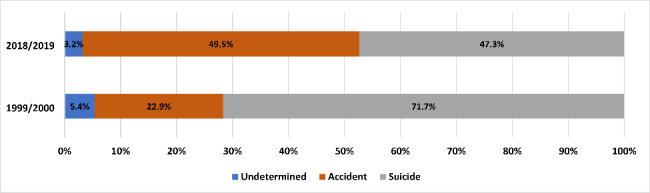
Percentage composition of self-injury mortality (SIM) by manner of death, 1999/2000 vs. 2018/2019 Annual-averaged SIM for each period is operationalized as a composite of suicides by any method, and 80% of drug overdose deaths among the population aged 15 years and older whose manner is accident and 90% of corresponding undetermined (intent) deaths.

Annual-averaged SIM deaths totaled 40,834 and 101,325 in 1999/2000 and 2018/2019, respectively (Tables 1 and 2)—including corresponding suicides of 29,275 and 47,928 (Tables 3 and 4). Figure 1 depicts pronounced changes in the proportional shares of the suicide and ‘accidental’/unintentional drug self-intoxication components of SIM between 1999/2000 and 2018/2019. By the end of the observation period, ‘accidental’ drug overdose deaths comprised approximately half of the total versus less than one-quarter at inception. Whereas the suicide share of SIM declined by a third, the suicide rate increased by 40% over the observation period (derived from Tables 3 and 4). Relatively small at both data points, the share for overdose deaths of undetermined intent declined by approximately 40%.

Estimated annual-averaged national costs of SIM rose by 143% from $0.46 trillion in 1999/2000 to $1.12 trillion in 2019 US dollars in 2018/2019, as the crude SIM rate increased by 112%—from 14.6 to 30.9 per 100,000 population (Tables 1 and 2). Cost of future quality of life losses predominated in both periods, but those of work losses also far exceeded medical spending. Respective ratios of quality of life and work losses to medical spending in 2019 dollars were 1,419 and 526 at the beginning of the observation period and 1,476 and 526 at its conclusion. National suicide costs increased 58%—from $318.6 billion to $502.7 billion (Tables 3 and 4). Respective quality of life and work losses to medical spending ratios were 1,489 and 544 and 1,585 and 552. National per capita costs of SIM doubled from $1,638 to $3,413 between 1999/2000 and 2018/2019 (Tables 1 and 2), whereas the costs of the suicide component rose 35%—from $1,137 to $1,534 (Tables 3 and 4).

**Fig. 2:**
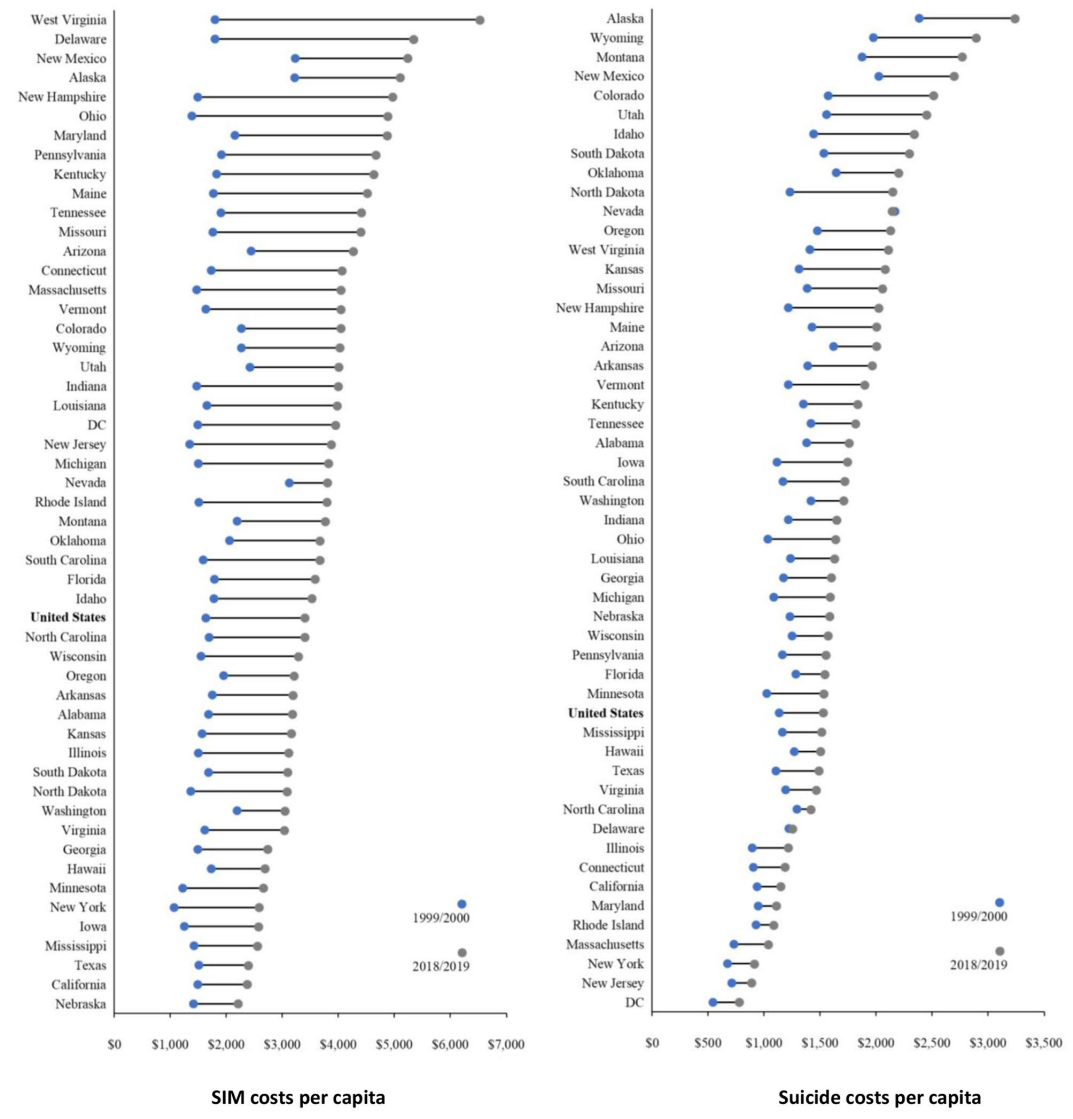
Ranked self-injury mortality (SIM) and suicide per capita costs by state, 2018/2019 vs. 1999/2000

Depicted in lollipop graphs (Fig. 2), states are ranked by per capita SIM and suicide costs at the end of the observation period; their comparative data for the beginning of the period are also shown. Specifics are reported in Table 5. First-ranked West Virginia was a clear outlier on SIM in 2018/2019 with a per capita cost of $6,534, and it ranked 13th on per capita suicide cost at $2,113. Delaware ranked second on per capita SIM cost at $5,351 and 42nd on the suicide metric at $1,256. Nebraska posted the lowest per capita SIM cost at $2,215 and ranked 32nd on per capita suicide cost at $1,591. Alaska posted the highest per capita suicide cost at $3,245, followed by Wyoming at $2,895, with DC the lowest at $781.

**Fig. 3:**
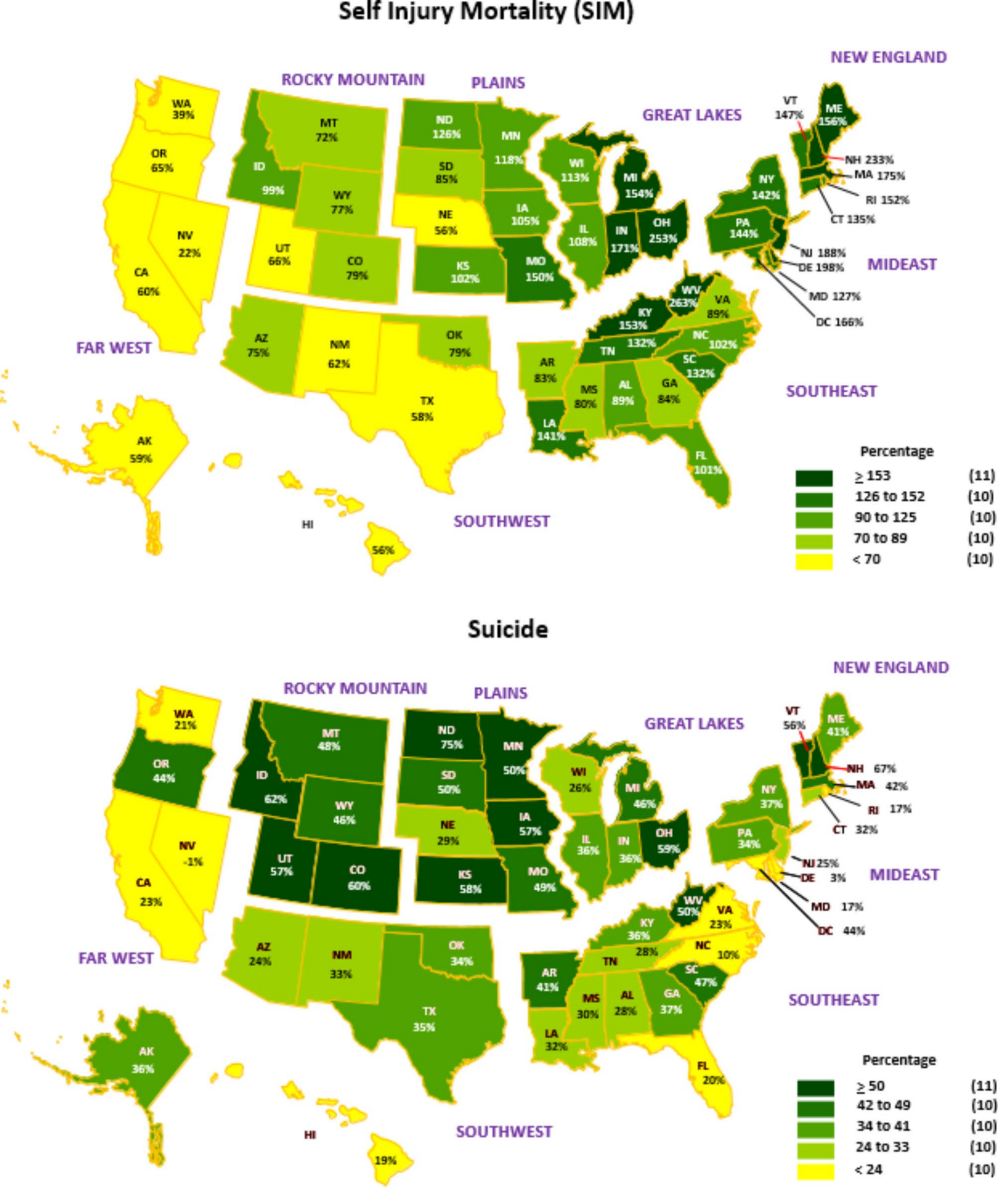
Percentage increase in self-injury mortality (SIM) and suicide costs per capita by region and state, 1999/2000—2018/2019

National chloropeth maps display the inter-period growth in per capita SIM and suicide costs by state and region, expressed in quintiles (Fig. 3). States in the top quintile for SIM, those whose per capita cost increases exceeded 152%, concentrated in the Great Lakes, Southeast, Mideast and New England regions. States in the bottom quintile, those with per capita cost increases below 70%, were located in the Far West, Southwest, Plains, and Rocky Mountain regions. West Virginia exhibited the largest increase at 263% and Nevada the smallest at 22%. Percentage per capita cost increases for suicide were smaller than for SIM. Only the Far West, Southwest and Mideast were not represented in the top quintile, which comprised states with increases of 50% or greater. The bottom quintile comprised states with per capita suicide cost increases below 24%. Regions represented were the Far West, Southeast, Mideast and New England. North Dakota and Nevada occupied the extremes on the cost change continuum at 75% and − 1%, respectively.

**Table 1:** Annual-averaged self-injury mortality (SIM) counts, rates and costs by state, 1999/2000

State	Mortality			Costs^a^					State		Mortality				Costs^a^				
	count	rate	medical		work loss	quality of life loss	total			count		rate	medical			work loss	quality of life loss	total	
**Alabama**	684	15.4	3.6		1,996.3	5,494.7	7,494.6		**Montana**	184		20.4	0.8			521.9	1,447.2	1,969.9	
**Alaska**	160	25.5	0.5		554.9	1,459.5	2,015.0		**Nebraska**	212		12.4	1.3			657.9	1,758.6	2,417.8	
**Arizona**	1,115	22.0	8.6		3,351.6	9,024.0	12,384.2		**Nevada**	562		28.6	2.2			1,642.2	4,506.0	6,150.5	
**Arkansas**	425	16.0	2.6		1,249.7	3,412.2	4,664.6		**New Hampshire**	162		13.2	0.7			501.6	1,331.3	1,833.6	
**California**	4,590	13.6	24.9		13,375.2	36,695.6	50,095.7		**New Jersey**	985		11.7	5.5			3,103.6	8,205.9	11,315.0	
**Colorado**	839	19.7	4.2		2,631.6	7,030.8	9,666.6		**New Mexico**	499		27.5	2.8			1,623.6	4,236.2	5,862.6	
**Connecticut**	515	15.2	2.8		1,614.9	4,273.6	5,891.3		**New York**	1,788		9.4	10.6			5,508.3	14,720.6	20,239.5	
**Delaware**	120	15.4	0.7		385.6	1,014.3	1,400.6		**North Carolina**	1,191		14.9	7.2			3,647.2	9,859.4	13,513.9	
**DC**	75	13.2	0.6		229.7	619.5	849.8		**North Dakota**	78		12.2	0.5			238.2	639.5	878.1	
**Florida**	2,715	17.1	15.1		7,456.4	20,900.7	28,372.2		**Ohio**	1,429		12.6	8.4			4,225.0	11,483.1	15,716.6	
**Georgia**	1,074	13.2	6.2		3,256.9	8,846.8	12,109.9		**Oklahoma**	617		17.9	4.1			1,936.2	5,159.0	7,099.4	
**Hawaii**	184	15.2	1.0		575.5	1,523.0	2,099.5		**Oregon**	621		18.2	3.1			1,766.4	4,890.4	6,659.9	
**Idaho**	211	16.4	1.2		604.6	1,680.9	2,286.7		**Pennsylvania**	2,066		16.8	11.2			6,426.3	17,092.1	23,529.6	
**Illinois**	1,608	13.0	8.8		5,105.7	13,443.4	18,557.8		**Rhode Island**	135		12.9	0.6			427.9	1,146.0	1,574.5	
**Indiana**	785	12.9	5.4		2,450.4	6,498.2	8,954.0		**South Carolina**	569		14.3	3.1			1,693.9	4,632.6	6,329.6	
**Iowa**	332	11.4	1.8		989.0	2,678.7	3,669.5		**South Dakota**	109		14.4	0.6			350.1	914.1	1,264.8	
**Kansas**	369	13.7	2.5		1,146.3	3,058.5	4,207.3		**Tennessee**	959		16.9	6.8			2,901.9	7,873.6	10,782.3	
**Kentucky**	653	16.2	4.2		2,009.7	5,364.1	7,378.0		**Texas**	2,721		13.1	17.3			8,519.4	22,835.6	31,372.3	
**Louisiana**	645	14.5	4.0		2,011.6	5,372.6	7,388.2		**Utah**	442		19.9	2.4			1,499.9	3,871.1	5,373.3	
**Maine**	199	15.6	0.9		613.6	1,633.1	2,247.7		**Vermont**	91		15.0	0.3			269.5	724.1	993.9	
**Maryland**	966	18.3	4.7		3,145.6	8,209.1	11,359.4		**Virginia**	1,022		14.5	5.8			3,059.1	8,314.2	11,379.2	
**Massachusetts**	777	12.3	4.8		2,568.1	6,743.3	9,316.2		**Washington**	1,150		19.6	7.3			3,457.8	9,397.5	12,862.7	
**Michigan**	1,320	13.3	7.4		4,041.6	10,882.9	14,931.9		**West Virginia**	298		16.4	1.8			869.9	2,386.3	3,258.0	
**Minnesota**	522	10.7	2.7		1,653.2	4,345.6	6,001.5		**Wisconsin**	727		13.6	3.8			2,240.9	6,027.1	8,271.8	
**Mississippi**	362	12.8	2.3		1,084.9	2,955.8	4,043.0		**Wyoming**	104		21.0	0.5			293.3	826.0	1,119.9	
**Missouri**	874	15.7	5.1		2,667.6	7,172.0	9,844.7		**UNITED STATES**	40,834		14.6	235.8			124,152.5	334,610.6	458,998.9	

^**a**^Costs are in millions of dollars and subcategories may not sum to totals due to rounding.

**Table 2:** Annual-averaged self-injury mortality (SIM) counts, rates and costs by state, 2018/2019

State	Mortality			Costs^a^					State	Mortality			Costs^a^				
	count	rate	medical		work loss	quality of life loss	total			count	rate	medical		work loss	quality of life loss	total	
**Alabama**	1,410	28.8	8.8		4,087.4	11,499.3	15,595.6		**Montana**	371	34.8	1.5		1,039.8	2,978.5	4,019.8	
**Alaska**	313	42.7	1.5		1,015.9	2,737.4	3,754.8		**Nebraska**	398	20.6	1.8		1,105.9	3,171.0	4,278.7	
**Arizona**	2,825	39.1	16.0		8,108.9	22,736.0	30,860.9		**Nevada**	1,128	36.9	6.4		2,948.8	8,713.8	11,669.0	
**Arkansas**	867	28.7	4.2		2,548.8	7,105.9	9,658.9		**New Hampshire**	592	43.6	2.3		1,811.0	4,940.8	6,754.2	
**California**	8,920	22.6	45.5		24,179.4	69,776.9	94,001.8		**New Jersey**	3,037	34.1	16.3		9,223.4	25,288.9	34,528.7	
**Colorado**	2,057	35.9	11.0		6,139.5	17,041.9	23,192.3		**New Mexico**	994	47.4	4.9		2,886.3	8,111.3	11,002.4	
**Connecticut**	1,321	37.0	6.9		3,828.3	10,711.2	14,546.3		**New York**	4,565	23.4	25.3		13,276.0	37,179.1	50,480.4	
**Delaware**	444	45.7	2.8		1,401.8	3,788.4	5,193.1		**North Carolina**	3,150	30.2	18.2		9,447.2	26,114.5	35,580.0	
**DC**	281	39.9	2.1		678.2	2,109.2	2,789.4		**North Dakota**	202	26.5	1.1		630.3	1,719.0	2,350.4	
**Florida**	7,265	34.0	45.0		19,810.0	56,957.2	76,812.2		**Ohio**	5,028	43.0	30.4		15,179.7	41,955.0	57,165.1	
**Georgia**	2,640	25.0	13.5		7,567.0	21,395.5	28,975.9		**Oklahoma**	1,331	33.7	6.9		3,776.4	10,759.0	14,542.3	
**Hawaii**	371	26.2	2.9		974.9	2,854.6	3,832.4		**Oregon**	1,285	30.6	5.5		3,469.4	10,043.8	13,518.7	
**Idaho**	577	32.6	2.9		1,624.2	4,635.8	6,262.8		**Pennsylvania**	5,341	41.7	26.6		15,873.1	44,041.8	59,941.5	
**Illinois**	3,577	28.1	20.5		10,406.2	29,221.4	39,648.2		**Rhode Island**	366	34.6	2.6		1,064.0	2,963.5	4,030.1	
**Indiana**	2,357	35.1	14.7		7,134.8	19,735.6	26,885.2		**South Carolina**	1,698	33.2	9.3		4,940.4	13,873.6	18,823.3	
**Iowa**	738	23.4	4.8		2,151.3	5,983.2	8,139.4		**South Dakota**	234	26.5	0.9		732.7	2,009.1	2,742.7	
**Kansas**	816	28.0	4.6		2,441.1	6,770.7	9,216.4		**Tennessee**	2,711	39.9	17.1		7,846.8	22,213.7	30,077.6	
**Kentucky**	1,845	41.3	10.2		5,445.4	15,254.5	20,710.1		**Texas**	6,236	21.6	37.1		18,246.1	50,965.1	69,248.3	
**Louisiana**	1,658	35.6	9.4		4,900.1	13,644.5	18,554.0		**Utah**	1,098	34.5	5.2		3,414.5	9,359.9	12,779.5	
**Maine**	559	41.7	1.9		1,596.2	4,471.5	6,069.7		**Vermont**	230	36.7	1.0		663.2	1,867.5	2,531.7	
**Maryland**	2,667	44.1	14.1		7,724.9	21,740.7	29,479.7		**Virginia**	2,327	27.3	12.6		6,861.6	19,109.6	25,983.9	
**Massachusetts**	2,445	35.4	12.4		7,479.6	20,443.2	27,935.2		**Washington**	2,163	28.6	12.4		5,953.5	17,164.0	23,130.0	
**Michigan**	3,467	34.7	18.7		9,990.2	28,265.3	38,274.2		**West Virginia**	1,036	57.6	5.4		3,117.1	8,632.3	11,754.9	
**Minnesota**	1,333	23.7	7.8		3,978.4	11,050.1	15,036.4		**Wisconsin**	1,727	29.7	10.0		5,015.9	14,114.0	19,139.8	
**Mississippi**	705	23.7	3.1		1,991.6	5,654.7	7,649.3		**Wyoming**	218	37.7	1.1		605.6	1,724.0	2,330.6	
**Missouri**	2,402	39.2	11.1		7,166.3	19,886.2	27,063.6		**UNITED STATES**	101,325	30.9	558.4		293,499.3	824,484.0	1,118,541.7	

^**a**^Costs are in millions of dollars and subcategories may not sum to totals due to rounding.

**Table 3:** Annual-averaged suicide counts, rates and costs by state, 1999/2000

	Mortality		Costs^a^					Mortality		Costs^a^			
State	count	rate per 100,000	medical	work loss	quality of	total	State	count	rate per 100,000	medical	work loss	quality of	total
					life loss							life loss	
**Alabama**	569	12.8	2.5	1,634.2	4,496.7	6,133.3	**Montana**	160	17.8	0.7	447.4	1,241.3	1,689.4
**Alaska**	117	18.6	0.4	417.4	1,075.0	1,492.8	**Nebraska**	185	10.8	1.0	574.1	1,531.4	2,106.6
**Arizona**	775	15.3	5.5	2,180.6	6,046.5	8,232.6	**Nevada**	402	20.4	1.5	1,129.9	3,128.7	4,260.1
**Arkansas**	343	12.9	1.9	989.4	2,710.6	3,701.9	**New Hampshire**	134	10.9	0.5	404.2	1,088.3	1,493.0
**California**	3,023	9.0	15.3	8,292.9	23,243.2	31,551.4	**New Jersey**	562	6.7	2.9	1,585.7	4,396.7	5,985.4
**Colorado**	594	13.9	2.6	1,815.8	4,880.1	6,698.5	**New Mexico**	323	17.8	1.6	1,001.1	2,672.8	3,675.5
**Connecticut**	289	8.5	1.5	812.0	2,262.7	3,076.2	**New York**	1,164	6.1	6.2	3,411.0	9,298.7	12,715.9
**Delaware**	84	10.8	0.5	261.8	691.5	953.8	**North Carolina**	928	11.6	5.3	2,780.6	7,569.8	10,355.7
**DC**	27	4.6	0.2	85.3	224.3	309.8	**North Dakota**	71	11.0	0.4	213.9	576.5	790.8
**Florida**	2,058	13.0	10.8	5,240.2	15,114.5	20,365.4	**Ohio**	1,095	9.7	6.1	3,138.2	8,606.9	11,751.2
**Georgia**	860	10.6	4.6	2,558.0	6,971.9	9,534.5	**Oklahoma**	495	14.4	3.2	1,545.1	4,113.4	5,661.7
**Hawaii**	137	11.3	0.8	423.8	1,113.2	1,537.9	**Oregon**	486	14.2	2.2	1,317.8	3,713.8	5,033.7
**Idaho**	174	13.5	0.9	491.9	1,363.4	1,856.1	**Pennsylvania**	1,320	10.8	6.5	3,823.8	10,447.9	14,278.2
**Illinois**	1,012	8.2	5.4	3,004.2	8,105.9	11,115.5	**Rhode Island**	86	8.2	0.4	264.0	704.6	969.0
**Indiana**	656	10.8	4.1	2,017.1	5,362.6	7,383.7	**South Carolina**	431	10.8	2.1	1,240.8	3,433.6	4,676.4
**Iowa**	297	10.2	1.5	878.4	2,375.9	3,255.8	**South Dakota**	99	13.2	0.5	321.3	834.5	1,156.3
**Kansas**	312	11.6	2.1	960.2	2,566.7	3,529.0	**Tennessee**	728	12.9	4.8	2,163.6	5,881.2	8,049.6
**Kentucky**	496	12.3	3.1	1,475.0	3,966.7	5,444.7	**Texas**	2,029	9.8	11.9	6,179.8	16,682.7	22,874.5
**Louisiana**	493	11.0	2.9	1,495.5	4,017.0	5,515.4	**Utah**	290	13.1	1.6	954.6	2,500.9	3,457.2
**Maine**	165	12.9	0.7	489.3	1,323.2	1,813.2	**Vermont**	70	11.5	0.2	197.6	539.2	737.1
**Maryland**	455	8.6	2.2	1,340.1	3,655.0	4,997.2	**Virginia**	780	11.1	4.1	2,216.2	6,165.5	8,385.8
**Massachusetts**	409	6.5	2.0	1,240.1	3,382.5	4,624.6	**Washington**	772	13.1	4.6	2,222.4	6,105.8	8,332.8
**Michigan**	974	9.8	4.7	2,919.5	7,880.1	10,804.3	**West Virginia**	237	13.1	1.3	676.1	1,870.4	2,547.8
**Minnesota**	439	9.0	1.9	1,382.9	3,635.8	5,020.7	**Wisconsin**	592	11.1	2.7	1,806.8	4,869.1	6,678.6
**Mississippi**	299	10.5	1.9	885.2	2,410.1	3,297.1	**Wyoming**	91	18.4	0.4	256.0	718.5	974.9
**Missouri**	700	12.5	3.8	2,075.7	5,638.0	7,717.5	**UNITED STATES**	29,275	10.4	156.6	85,238.4	233,205.4	318,600.3

^a^ Costs in millions of dollars and sub-categories may not sum to totals due to rounding.

**Table 4:** Annual-averaged suicide counts, rates and costs by state, 2018/2019

	Mortality		Costs^a^					Mortality		Costs^a^			
State	count	rate per 100,000	medical	work loss	quality of	total	State	count	rate per 100,000	medical	work loss	quality of	total
					life loss							life loss	
**Alabama**	814	16.6	4.9	2,249.2	6,378.7	8,632.3	**Montana**	277	26.0	0.9	761.8	2,192.2	2,954.8
**Alaska**	197	26.8	0.9	652.0	1,730.7	2,383.5	**Nebraska**	290	15.0	1.2	799.9	2,272.3	3,073.4
**Arizona**	1,429	19.8	6.8	3,725.6	10,774.5	14,506.9	**Nevada**	650	21.2	3.0	1,666.7	4,896.5	6,566.2
**Arkansas**	551	18.3	2.2	1,557.5	4,371.7	5,931.4	**New Hampshire**	267	19.7	0.9	705.1	2,047.9	2,753.9
**California**	4,464	11.3	20.2	11,659.4	33,836.9	45,516.5	**New Jersey**	770	8.7	4.0	2,009.6	5,917.6	7,931.2
**Colorado**	1,297	22.6	6.4	3,805.9	10,589.6	14,402.0	**New Mexico**	525	25.0	2.4	1,473.6	4,178.6	5,654.7
**Connecticut**	427	12.0	2.0	1,059.2	3,195.2	4,256.5	**New York**	1,714	8.8	8.4	4,589.7	13,280.2	17,878.3
**Delaware**	112	11.5	0.5	318.5	899.6	1,218.6	**North Carolina**	1,426	13.7	7.0	3,804.3	11,017.1	14,828.4
**DC**	49	7.0	0.3	143.9	405.8	550.0	**North Dakota**	142	18.6	0.7	439.0	1,199.4	1,639.1
**Florida**	3,516	16.4	18.9	8,079.3	24,939.5	33,037.7	**Ohio**	1,822	15.6	10.9	4,978.6	14,205.5	19,194.9
**Georgia**	1,577	14.9	7.3	4,437.7	12,527.9	16,972.9	**Oklahoma**	803	20.3	3.4	2,285.8	6,415.2	8,704.4
**Hawaii**	200	14.1	1.2	563.4	1,571.6	2,136.2	**Oregon**	875	20.8	3.1	2,282.4	6,685.7	8,971.2
**Idaho**	391	22.1	1.8	1,076.5	3,072.2	4,150.5	**Pennsylvania**	1,955	15.3	8.7	5,071.0	14,834.3	19,914.0
**Illinois**	1,464	11.5	7.5	4,004.6	11,484.2	15,496.3	**Rhode Island**	115	10.8	0.7	291.1	860.0	1,151.8
**Indiana**	1,026	15.3	5.2	2,909.9	8,186.9	11,102.0	**South Carolina**	832	16.3	4.2	2,305.8	6,521.1	8,831.1
**Iowa**	509	16.1	3.0	1,459.4	4,059.3	5,521.8	**South Dakota**	175	19.8	0.6	543.2	1,489.0	2,032.8
**Kansas**	540	18.5	2.7	1,624.3	4,441.4	6,068.5	**Tennessee**	1,190	17.5	6.5	3,171.4	9,185.7	12,363.6
**Kentucky**	778	17.4	3.6	2,132.1	6,076.6	8,212.3	**Texas**	3,911	13.6	21.9	11,392.6	31,702.0	43,116.6
**Louisiana**	712	15.3	3.5	1,979.2	5,605.7	7,588.4	**Utah**	660	20.7	2.8	2,124.2	5,682.5	7,809.5
**Maine**	273	20.4	1.0	678.9	2,014.9	2,694.8	**Vermont**	118	18.8	0.4	301.2	886.2	1,187.9
**Maryland**	654	10.8	3.0	1,720.0	5,002.9	6,725.8	**Virginia**	1,192	14.0	6.3	3,224.7	9,286.6	12,517.5
**Massachusetts**	694	10.1	3.1	1,816.5	5,349.6	7,169.1	**Washington**	1,258	16.6	5.8	3,317.5	9,665.2	12,988.6
**Michigan**	1,510	15.1	7.7	4,132.7	11,792.9	15,933.4	**West Virginia**	363	20.2	1.7	987.1	2,811.8	3,800.6
**Minnesota**	785	13.9	4.1	2,282.0	6,353.3	8,639.4	**Wisconsin**	867	14.9	4.4	2,375.3	6,788.5	9,168.2
**Mississippi**	429	14.4	1.7	1,178.6	3,338.5	4,518.8	**Wyoming**	159	27.4	0.7	437.4	1,236.1	1,674.2
**Missouri**	1,186	19.3	4.9	3,298.5	9,344.8	12,648.2	**UNITED STATES**	47,928	14.6	235.1	129,883.8	372,602.0	502,720.9

^a^ Costs in millions of dollars and sub-categories may not sum to totals due to rounding.

**Table 5:** Self-injury mortality (SIM) and suicide costs per capita by state, 1999/2000 and 2018/2019

State	SIM cost per capita			suicide cost per capita			State	SIM cost per capita				suicide cost per capita			
	1999/2000	2018/2019	% change from 1999/2000 to 2018/2019	1999/2000	2018/2019	% change from 1999/2000 to 2018/2019		1999/2000	2018/2019	% change from 1999/2000 to 2018/2019	1999/2000		2018/2019	% change from 1999/2000 to 2018/2019	
**Alabama**	$1,689	$3,186	89%	$1,382	$1,763	28%	**Montana**	$2,189	$3,773	72%	$1,877		$2,773	48%	
**Alaska**	$3,220	$5,112	59%	$2,385	$3,245	36%	**Nebraska**	$1,416	$2,215	56%	$1,233		$1,591	29%	
**Arizona**	$2,439	$4,271	75%	$1,621	$2,008	24%	**Nevada**	$3,128	$3,817	22%	$2,166		$2,148	-1%	
**Arkansas**	$1,752	$3,203	83%	$1,390	$1,967	41%	**New Hampshire**	$1,492	$4,973	233%	$1,215		$2,028	67%	
**California**	$1,487	$2,378	60%	$937	$1,151	23%	**New Jersey**	$1,349	$3,882	188%	$714		$892	25%	
**Colorado**	$2,267	$4,050	79%	$1,571	$2,515	60%	**New Mexico**	$3,233	$5,249	62%	$2,027		$2,698	33%	
**Connecticut**	$1,735	$4,076	135%	$906	$1,193	32%	**New York**	$1,069	$2,589	142%	$672		$917	37%	
**Delaware**	$1,797	$5,351	198%	$1,224	$1,256	3%	**North Carolina**	$1,689	$3,409	102%	$1,295		$1,421	10%	
**DC**	$1,488	$3,962	166%	$542	$781	44%	**North Dakota**	$1,365	$3,088	126%	$1,229		$2,154	75%	
**Florida**	$1,788	$3,591	101%	$1,283	$1,545	20%	**Ohio**	$1,385	$4,890	253%	$1,036		$1,642	59%	
**Georgia**	$1,492	$2,742	84%	$1,175	$1,606	37%	**Oklahoma**	$2,061	$3,682	79%	$1,644		$2,204	34%	
**Hawaii**	$1,734	$2,702	56%	$1,270	$1,506	19%	**Oregon**	$1,954	$3,216	65%	$1,477		$2,134	44%	
**Idaho**	$1,780	$3,537	99%	$1,445	$2,344	62%	**Pennsylvania**	$1,917	$4,681	144%	$1,163		$1,555	34%	
**Illinois**	$1,498	$3,120	108%	$897	$1,220	36%	**Rhode Island**	$1,508	$3,808	153%	$928		$1,088	17%	
**Indiana**	$1,477	$4,006	171%	$1,218	$1,654	36%	**South Carolina**	$1,585	$3,679	132%	$1,171		$1,726	47%	
**Iowa**	$1,256	$2,579	105%	$1,114	$1,750	57%	**South Dakota**	$1,681	$3,105	85%	$1,536		$2,301	50%	
**Kansas**	$1,568	$3,165	102%	$1,315	$2,084	58%	**Tennessee**	$1,904	$4,423	132%	$1,421		$1,818	28%	
**Kentucky**	$1,831	$4,635	153%	$1,351	$1,838	36%	**Texas**	$1,515	$2,400	58%	$1,105		$1,495	35%	
**Louisiana**	$1,655	$3,986	141%	$1,235	$1,630	32%	**Utah**	$2,422	$4,014	66%	$1,558		$2,453	57%	
**Maine**	$1,769	$4,525	156%	$1,427	$2,009	41%	**Vermont**	$1,638	$4,050	147%	$1,215		$1,900	56%	
**Maryland**	$2,153	$4,877	127%	$947	$1,113	17%	**Virginia**	$1,617	$3,047	89%	$1,191		$1,468	23%	
**Massachusetts**	$1,471	$4,050	175%	$730	$1,039	42%	**Washington**	$2,192	$3,053	39%	$1,420		$1,715	21%	
**Michigan**	$1,506	$3,831	154%	$1,089	$1,595	46%	**West Virginia**	$1,800	$6,534	263%	$1,408		$2,113	50%	
**Minnesota**	$1,226	$2,673	118%	$1,025	$1,536	50%	**Wisconsin**	$1,547	$3,290	113%	$1,249		$1,576	26%	
**Mississippi**	$1,425	$2,566	80%	$1,162	$1,516	30%	**Wyoming**	$2,273	$4,030	77%	$1,978		$2,895	46%	
**Missouri**	$1,765	$4,414	150%	$1,383	$2,063	49%	**UNITED STATES**	$1,638	$3,413	108%	$1,137		$1,534	35%	

## Discussion

The magnitude and escalation of economic costs of self-injury mortality (SIM) over the past two decades have been monumental when viewed both through the lens of the nation and states. Given the burgeoning suicide and drug overdose epidemics during the study period [8], estimated SIM costs escalated markedly for the US as a whole and by state and region: total costs in 2019 dollars more than doubled from $0.46 trillion to $1.12 trillion. The indirect costs of work and future quality of life losses eclipsed medical expenditures. National annual average per capita costs of SIM at the end of the observation period were $3,413 versus $1,638 at the beginning. Perennially economically depressed and the only state fully immersed in Appalachia, West Virginia was a clear outlier in 2018/2019 with a per capita cost of $6,534. Nebraska posted the lowest per capita SIM cost at $2,215. Total national SIM costs increased 2.5 times more than those for suicide, with the latter reaching half a trillion dollars in 2018/2019, a 58% rise since 1999/2000. DC and Alaska, respectively, posted the lowest and highest per capita costs of suicide at both observation points. Regionally, the largest cost increases for SIM mainly concentrated in the eastern half of the US, whereas corresponding cost increases for suicide were more dispersed.

A secular increase in life expectancy at birth in the US characterized most of the 20th century and through 2014 [42]. However, preceding the epidemiologic and demographic devastation now being wreaked by the COVID-19 pandemic, life expectancy manifested a modest (0.3 years) but unprecedented decline over the next three years [43]. Although mainly implicating the ‘deaths of despair,’ we hypothesize that the SIM component, rather than the chronic disease component, is the more potent catalyst of this pre-COVID-19 reversal in life expectancy due to the disproportionally greater SIM attrition occurring among younger and middle-aged adults. Indeed, the premature mortality that typifies SIM [13] elevates its importance as a public health problem beyond the mere numbers. While deaths of despair were initially most pronounced among non-Hispanic Whites, particularly middle-aged men [11], larger relative increases in SIM rates have since been observed among non-Hispanic Blacks, Hispanics and women [22]. Thus, as with COVID-19, the universality of SIM is not only geographic.

Our primary motivation for conceiving and implementing SIM was the profound undercounting of suicides among drug-related fatalities [14, 15], the nonrandom nature of misclassification across methods/injury mechanisms, and the likelihood they have differential impacts related to age, sex, race/ethnicity, educational attainment and psychiatric history [17, 22]. Whereas SIM necessarily serves to approximate the fatal impact of self-injurious behaviors, it is a needed adjustment to accommodate the relative paucity of in-depth postmortem investigations conducted in many regions of the US [44], a problem likely exacerbated during the second decade of the 21st century due to the heavy burden and diminished resources associated with surging fentanyl-related opioid deaths [45–47]. Notably, we restricted SIM to drug-related fatalities even when one might argue for inclusion of other causes of death, such as motor vehicle crashes associated with high-risk behaviors [13]. However, identifying the latter cases with confidence using currently available death records is infeasible, which implies our estimates of total costs might be lower than the full impact associated with self-harm injury fatalities.

It warrants emphasizing that the magnitude and escalation of the economic costs of SIM during the two decades of our study were enormous—now exceeding a trillion dollars annually. While the rising costs have been driven more by drug fatalities formally categorized as accidents and undetermined deaths, the annual cost of suicides is approximately half a trillion dollars. Suicide and drug fatalities occur in populations with overlapping risk factors; for example, in the US one in four suicides involves alcohol consumption and one in five suicides opioid consumption [48]. Furthermore, two studies of treatment-seeking inpatients with a history of non-fatal opioid overdose found that, just prior to their most recent overdose event, nearly half reported some desire to die and one in five reported some intention to die [49, 50]. The FY2022 Federal budget to address SIM-related conditions, including appropriations for CDC, the Substance Abuse and Mental Health Services Administration, and the National Institutes of Health, approaches $5.3 billion; of this total, nearly $262 million is clearly devoted to suicide prevention and research, with the remainder largely focused on drug-related interventions, prevention, and research [51, 52].

Current prevention initiatives emphasize downstream measures, such as identifying, counseling, and treating high-risk individuals in clinical, educational, work, criminal justice and other institutional settings. However, there are insufficient resources to meet the current clinical needs of persons challenged by co-occurring mental health and substance use disorders. Moreover, without addressing upstream factors—such as an unstable economic environment and social inequity [53]—there will be no diminution of people needing prevention and treatment services. The nation now faces the challenge of either directly financing the costs for preventing and treating persons suffering mental health and substance disorders or later incurring the much larger, but less visible, costs, and familial and community distress and sorrow, including increased risk of additional suicides among friends and family, tied to the prodigious loss of lives they inflict. Given that most Federal funds are distributed to states by block grants and by selective grants to local governmental agencies, implementation of both upstream and downstream prevention programs depends on engaged local and state implementation. Without recognition of the enormous financial and social costs to states and communities, as highlighted by our findings, there is the continuing threat that well-designed prevention initiatives will remain underfunded.

### Limitations

Our measurement of the ‘nonsuicide’ drug component of SIM is indirect. More direct measurement would be attainable with the incorporation of a self-injury checkbox on the death certificate [54]. In a given drug-intoxication death, whose manner was classified by a medicolegal official as ‘accident’ or undetermined intent, a self-injury designation would be justifiable with such corroborative evidence as the recording of drug paraphernalia at the death scene; proof of doctor and/or pharmacy shopping identified through prescription drug monitoring programs; and the determination of a non-therapeutic dose of prescription drugs or use of lethal illicit drugs determined by toxicologic testing. While we envisage the expansion of SIM beyond suicides and the drug component, to include other deaths like select motor vehicular trauma deaths [13], with the added complexity extant data systems and linkage makes this infeasible in the foreseeable future. Our application of SIM is pragmatic and conservative, but likely reflects the great preponderance of the injury deaths of despair.

The cost estimates in this study or any similar effort are inherently indirect and approximate. As well as medical costs and work losses, we factored in quality-of-life losses by monetizing the estimated quality-adjusted life years lost using CDC’s WISQARS Cost of Injury Module, which facilitates comparisons with other estimates [24]. Our estimates neither captured collateral injuries to others, such as murder-suicide, nor incorporated some non-health costs, such as criminal justice costs related to death scene response/investigation and the adverse legal implications of decedents’ substance acquisition and misuse; and toxic stress and educational impacts on the decedent’s children; and damage to property [2]. Although most US government agencies use an estimated VSL in the range we used, numerous meta-analyses find confidence limits approximating plus or minus 40% around that estimate [55]. This study did not factor in inter-period volatility in SIM trends, exemplified by an accelerated rate [8] emanating from the spread of illicit fentanyl-related drugs [16]. Moreover, it does not include increased opioid deaths that occurred from 2019 to 2021 across the country, especially in Western states [56, 57]. Heterogeneity in medicolegal death investigations systems may have depressed SIM costs. A recent multivariable study found that states with a medical examiner system under centralized authority had higher adjusted SIM rates than other states [53]. This finding suggests that offices with superior staff training and experience, equipment, technical rigor, and scope in the conduct of death investigations detect more cases.

A more fundamental limitation arises from our exclusive use of mortality figures; estimating costs for those who have not died but suffer the conditions antecedent to death was beyond the scope of our current work. Associated with the onset of COVID-19, the mortality rate for opioid-related drug use continued to increase sharply during 2020 [58] and mean life expectancy at birth plummeted an estimated 1.87 years (credible range: 1.70–2.03) [59], indicating our cost estimates are low. We excluded the 2020 data from our study because of the intersecting nature of SIM and COVID-19 deaths and the unmet need to couple them with 2021 unit record data to stabilize state-based cost estimates. The latter data will not be released by CDC/National Center for Health Statistics until early 2023. Although our estimate for total annualized costs of SIM already exceeds $1 trillion, we are aware there will be a need for further studies to estimate the impact of the pandemic and the continuing influx and distribution of especially lethal synthetic opioids. Finally, most cost-effectiveness studies do not calculate standard errors (or confidence intervals) of cost estimates due to lack of data on the covariation matrix of costs and health outcomes by risk factors or diseases and injuries. Our ability to compute standard errors was further limited by the lack of income data specific to people who died from SIM or suicide.

## Conclusion

The scale and surge of the economic costs of SIM to society are large. To be effective, efforts to prevent deaths arising from self-injurious behaviors cannot be confined to downstream measures alone; addressing upstream factors, such as an unstable economic environment and social inequity, must be part of comprehensive approaches. Federal and state appropriations to support prevention and intervention programs should be financed with a clear understanding of the total costs—fiscal, social, and personal—incurred from deaths due to self-injurious behaviors. If sustainable interventions can be devised and implemented, periodic monitoring and evaluation will be essential.

## Data Availability

The suite of SAS code and costing factor datasets for the national fatal injury cost model are available (without support or a user’s guide) to non-commercial users upon request. These programs operate on Multiple Cause of Death datasets publicly available from the US Centers for Disease Control and Prevention (CDC). CDC’s Wide-ranging ONline Data for Epidemiologic Research (WONDER) is available at https://wonder.cdc.gov/ucd-icd10.htmland the Web-based Injury Statistics Query and Reporting System (WISQARS) Cost of Injury Module at https://www.researchgate.net/profile/Ted-Miller-5/publication/265162679_Medical_and_Work_Loss_Cost_Estimation_Methods_for_the_WISQARS_Cost_of_Injury_Module/links/54013c660cf2c48563aef010/Medical-and-Work-Loss-Cost-Estimation-Methods-for-the-WISQARS-Cost-of-Injury-Module.pdf?origin=publication_detail.

## Abbreviations

SIM: Self-injury mortality
US: United States
DC: District of Columbia
CDC: Centers for Disease Control and Prevention
WONDER: Wide-ranging ONline Data for Epidemiologic Research
WISQARS: Web-based Injury Statistics Query and Reporting System
VSL: Value of a statistical life
HIV: Human immunodeficiency virus infection
COVID-19: Coronavirus Disease 2019
STROBE: Strengthening the Reporting of Observational Studies in Epidemiology reporting guideline
ICD-10: International Statistical Classification of Diseases and Related Health Problems, Tenth Revision
FY: Fiscal Year
